# Non-destructive phenotyping for early seedling vigor in direct-seeded rice

**DOI:** 10.1186/s13007-020-00666-6

**Published:** 2020-09-21

**Authors:** Annamalai Anandan, Anumalla Mahender, Rameswar Prasad Sah, Lotan Kumar Bose, Hatanath Subudhi, Jitendra Meher, Janga Nagi Reddy, Jauhar Ali

**Affiliations:** 1 Crop Improvement Division, Indian Council of Agricultural Research-National Rice Research Institute (ICAR-NRRI), Cuttack, Odisha 753006 India; 2 Rice Breeding Platform, International Rice Research Institute (IRRI), Los Baños, Laguna, 4031 Philippines

**Keywords:** Direct-seeded rice, Early seedling vigor, Imaging, Breeding

## Abstract

**Background:**

Early seedling vigor is an essential trait of direct-seeded rice. It helps the seedlings to compete with weeds for water and nutrient availability, and contributes to better seedling establishment during the initial phase of crop growth. Seedling vigor is a complex trait, and phenotyping by a destructive method limits the improvement of this trait through traditional breeding. Hence, a non-invasive, rapid, and precise image-based phenotyping technique is developed to increase the possibility to improve early seedling vigor through breeding in rice and other field crops.

**Results:**

To establish and assess the methodology using free-source software, early seedling vigor was estimated from images captured with a digital SLR camera in a non-destructive way. Here, the legitimacy and strength of the method have been proved through screening seven diverse rice cultivars varying for early seedling vigor. In the regression analysis, whole-plant area (WPA) estimated by destructive-flatbed scanner (WPAs) and non-destructive imaging (WPA_i_) approaches was strongly related (R^2^ > 83%) and suggested that WPA_i_ can be adapted in place of destructive methods to estimate seedling vigor. In addition, this study has identified a set of new geometric traits (convex hull and top view area) for screening breeding lines for early seedling vigor in rice, which decreased the time by 80% and halved the cost of labor in data observation.

**Conclusions:**

The method demonstrated here is affordable and easy to establish as a phenotypic platform. It is suitable for most glasshouses/net houses for characterizing genotypes to understand the plasticity of shoots under a given environment at the seedling stage. The methodology explained in this experiment has been proven to be practical and suggested as a technique for researchers involved in direct-seeded rice. Consequently, it will help in the simultaneous screening of genotypes in large numbers, the identification of donors, and in gaining information on the genetic basis of the trait to design a breeding program for direct-seeded rice.

## Background

The benefits of decreasing the water footprint along with less labor use and an increase in the cost–benefit ratio have led rice farmers to shift their puddled-transplanted rice to direct-seeded rice (DSR). For successful crop establishment under DSR, rapid uniform emergence and accumulation of biomass in the early phase of crop growth are the key factors [1]. Thus, understanding the spatio-temporal changes in shoot biomass in the early phase of the crop by imaging would help to differentiate lines for vigor and provide insight into the physiology of rice seedlings under direct-seeded conditions [2, 3]. Therefore, developing an automated non-destructive screening method for an essential agronomic trait would enhance the productivity of rice under direct-seeded conditions.

Non-destructive phenotyping techniques are the key factors for screening and developing suitable rice genotypes for the target environment in a brief period [4, 5]. Several phenotyping methods have been optimized for screening biotic and abiotic stresses [6]. The absence of a suitable non-destructive-based high-throughput phenotyping system has restricted the exploitation of agronomically important traits in rice. Accumulation of biomass in the early phase of crop growth is necessary under DSR and is considered an important parameter for seedling vigor [1, 7]. Therefore, a genotype with early seedling vigor has significance in smothering the effect of weed competitiveness and water use efficiency to maintain the sustainability of rice production in rainfed and DSR conditions. The key limiting factor in developing rice varieties for direct-seeded conditions with early seedling vigor is the non-availability of a suitable non-destructive phenotyping screening technique to select genotypes against weed competitiveness with a consistent result. As biomass/shoot weight was observed to be closely related to early vigor measured at 14 and 28 days after sowing (DAS) [7], screening genotypes by measuring biomass with a non-destructive method has added advantages over other methods.

The early phase of a crop is more fragile and dynamic in response to the environment, and the complex nature of the trait poses a problem in phenotyping for early seedling vigor [8]. Further, phenotyping by destructive sampling and collecting of seeds from the same individuals, in the case of segregating generations, would be an additional impossible target. Recent advances in genomic technologies have changed the way of breeding programs by generating more genotypic data. Nevertheless, the same breeding programs failed to achieve the objective when the translation of such data failed to identify genotypes with the desirable trait [9]. Therefore, to overcome this bottleneck and to use those genotypic data in an efficient way, non-destructive phenotyping with precision is highly valued. In recent years, several reports have presented the advantages of non-destructive phenotyping by imaging techniques using near-infrared reflectance and spectral imaging using fluorescence and thermal wavelengths [10–12]. The application of image-based phenotyping is picking up in the area of field crops to understand the complex traits that are highly influenced by the environment. Several screening protocols and pipelines for data analysis were developed for some of the intricate stress factors such as salinity, nitrogen, and water deficiency, and nodal root angle in barley, rice, and sorghum, etc. [3, 9, 13–20]. Conversely, imaging techniques for early seedling vigor have not been standardized in rice. Most of the protocols developed by imaging are automated and require high-end facilities. Irrespective of the countries possessing those high-end automated facilities, they are not affordable to all researchers.

The existing field-based screening methodology to estimate seedling vigor is based on harvesting samples over relative time [7], which is labor-intensive. As the early phase of seedlings is dynamic in nature and in collecting data to estimate growth analysis, biomass or leaf area index from a subset of the population would not provide reasonable information. However, whole-plant area (WPA) is associated with seedling vigor, but the destructive method cannot capture the actual area. Therefore, a phenotyping screening protocol needs to be developed in a cost-effective way that is easy to handle, less labor-intensive, suitable for screening year-round, and amenable to integrating those phenotypic data with genotypic data generated from forward genetic tools such as genome-wide association mapping, linkage mapping, and gene sequencing. In addition, a non-destructive image-based phenotyping protocol should be flexible and experimentally verified by comparing it with existing field-based techniques. On the other hand, high-throughput phenotyping integrated with imaging techniques would be more flexible to capture the dynamic changes taking place in plants over a time interval. This would decrease genotype x environment interaction and several parameters such as compactness, leaf rolling, and drying related to abiotic stress and leaf damage due to pests and diseases would be measured seamlessly [21].

In the present study, we focused on establishing a non-destructive phenotyping protocol to estimate early seedling vigor in rice using images. Seven rice (*Oryza sativa* L.) genotypes of improved and traditional lines were grown in a pot under normal conditions without stagnation of water. Growth rates and related agro-morphological traits of those genotypes were measured by proposed non-destructive image-based and conventional destructive harvests to test the protocol efficiency, reproducibility, and ability to differentiate vigorous genotypes.

## Results

### Plant growth and partitioning of biomass among genotypes at 14 and 28 DAS

Significant differences were observed among the seven genotypes for traits studied at 14 and 28 DAS. On the 14th day after sowing, 16 traits exhibited significant differences among the 28 traits studied, while 19 traits showed significant differences between genotypes at 28 DAS (Table 1). However, traits observed after manual sampling such as shoot length and leaf number per plant were found to be significant across the two dates of observation (Tables 1, 2). Third-leaf width, eccentricity, convex hull, caliper length, whole-plant area by destructive-flatbed scanner (WPA_s_), whole-plant area by non-destructive imaging (WPA_i_), top view area, and compactness exhibited strong significant differences across the two dates of observation (Tables 3, 4) and these differences were captured well by the images from all seven genotypes grown under net house conditions. This suggests that observing growth parameters by imaging could capture subtle differences across genotypes, which is not possible in the traditional way of screening genotypes by destructive sampling. Among the seven genotypes, GM-217 and Vandana registered higher values for growth parameters such as shoot length, leaf number, WPA_i_, convex hull, and compactness at 14 DAS. On the other hand, LB-46 and Varshadhan were observed to have maximum leaf number, WPA_i_, and convex hull at 28 DAS. Traits such as root length, shoot dry weight, root dry weight, tiller number, and stem weight displayed a significant difference at 28 DAS, whereas these traits displayed non-significant growth at 14 DAS.

**Table 1:** Growth parameters observed by manual sampling and ANOVA for seven rice genotypes at 14 days after sowing

Traits	LB-46	GM-217	AC38399	ARC10656	Vandana	Sabita	Varshadhan	ANOVA
Shoot length (cm)	24.22 ± 1.36	30.78 ± 0.83	23.40 ± 1.63	22.3 ± 1.19	27.76 ± 0.65	20.28 ± 0.64	22.82 ± 1.05	**
Root length (cm)	14.98 ± 0.28	15.34 ± 1.47	16.76 ± 0.78	16.26 ± 0.77	18.48 ± 1.27	17.24 ± 0.46	19.94 ± 3.73	NS
Shoot dry weight (g)	0.0466 ± 0.007	0.059 ± 0.008	0.0382 ± 0.005	0.033 ± 0.001	0.0528 ± 0.002	0.0342 ± 0.004	0.0996 ± 0.05	NS
Root dry weight (g)	0.0182 ± 0.002	0.0246 ± 0.006	0.0174 ± 0.002	0.020 ± 0.004	0.0212 ± 0.002	0.0192 ± 0.002	0.0146 ± 0.0015	NS
Seed weight with mesocotyl	0.0154 ± 0.002	0.0494 ± 0.03	0.0108 ± 0.006	0.0062 ± 0.001	0.0132 ± 0.001	0.0094 ± 0.005	0.01 ± 0.001	NS
Mesocotyl length (cm)	0.38 ± 0.04	0.34 ± 0.02	0.32 ± 0.03	0.30 ± 0.04	0.34 ± 0.02	0.30 ± 0.04	0.38 ± 0.03	NS
Stem thickness (mm)	1.47 ± 0.07	1.25 ± 0.02	1.14 ± 0.08	1.38 ± 0.06	1.40 ± 0.008	1.10 ± 0.06	1.32 ± 0.028	**
Leaf number/plant	3.6 ± 0.24	4.0 ± 0.31	3.0 ± 0.05	3.2 ± 0.2	3.0 ± 0.01	3.0 ± 0.001	3.6 ± 0.24	**
First leaf weight (g)	0.0032 ± 0.0004	0.0034 ± 0.0003	0.0030 ± 0.0004	0.0030 ± 0.0002	0.0048 ± 0.0003	0.0044 ± 0.003	0.0026 ± 0.0004	NS
Second leaf weight (g)	0.0088 ± 0.003	0.0078 ± 0.001	0.0086 ± 0.001	0.0058 ± 0.002	0.0108 ± 0.005	0.0068 ± 0.007	0.0064 ± 0.006	NS
Third leaf weight (g)	0.0094 ± 0.004	0.0098 ± 0.004	0.0096 ± 0.002	0.0078 ± 0.003	0.0168 ± 0.006	0.007 ± 0.005	0.0108 ± 0.004	*

Mean ± standard error and significance of ANOVA are presented for each variety

*NS* non-significant

* p < 0.05; ** p < 0.001

**Table 2:** Growth parameters observed by manual sampling and ANOVA for seven rice genotypes at 28 days after sowing

Traits	LB-46	GM-217	AC38399	ARC10656	Vandana	Sabita	Varshadhan	ANOVA
Shoot length (cm)	41.48 ± 2.76	49.52 ± 2.4	42.92 ± 1.76	43.68 ± 0.37	43.88 ± 2.05	39.76 ± 0.81	39.00 ± 1.77	*
Root length (cm)	25.32 ± 1.46	25.40 ± 1.48	28.22 ± 1.18	26.72 ± 1.42	25.78 ± 2.21	32.28 ± 1.67	23.44 ± 1.63	*
Shoot dry weight (g)	0.396 ± 0.033	0.303 ± 0.10	0.188 ± 0.036	0.136 ± 0.011	0.180 ± 0.026	0.159 ± 0.009	0.269 ± 0.039	**
Root dry weight (g)	0.27 ± 0.025	0.169 ± 0.05	0.142 ± 0.03	0.073 ± 0.011	0.149 ± 0.015	0.088 ± 0.007	0.112 ± 0.013	**
Tiller number	3.40 ± 0.24	1.80 ± 0.58	1.20 ± 0.20	1.00 ± 0.001	1.00 ± 0.001	1.20 ± 0.20	2.20 ± 0.37	**
Stem weight (g)	0.38 ± 0.17	0.15 ± 0.04	0.07 ± 0.01	0.07 ± 0.003	0.09 ± 0.02	0.07 ± 0.01	0.11 ± 0.01	*
Stem thickness (mm)	3.48 ± 0.31	3.46 ± 0.20	3.05 ± 0.20	2.81 ± 0.12	3.13 ± 0.17	3.12 ± 0.18	2.70 ± 0.19	NS
Leaf number/plant	11.2 ± 1.16	7.40 ± 1.40	6.40 ± 0.68	6.40 ± 0.24	6.20 ± 0.20	6.20 ± 0.20	8.80 ± 1.24	**
First leaf weight (g)	0.002 ± 0.0001	0.003 ± 0.0004	0.003 ± 0.0002	0.003 ± 0.0004	0.003 ± 0.0002	0.005 ± 0.0006	0.003 ± 0.0002	NS
Second leaf weight (g)	0.008 ± 0.0009	0.006 ± 0.0008	0.004 ± 0.0006	0.006 ± 0.0009	0.006 ± 0.0008	0.010 ± 0.0002	0.007 ± 0.0009	NS
Third leaf weight (g)	0.010 ± 0.001	0.011 ± 0.002	0.005 ± 0.001	0.011 ± 0.001	0.012 ± 0.002	0.011 ± 0.001	0.014 ± 0.002	NS
Fourth leaf weight (g)	0.013 ± 0.002	0.017 ± 0.002	0.016 ± 0.002	0.018 ± 0.001	0.024 ± 0.002	0.017 ± 0.001	0.023 ± 0.002	NS
Fifth leaf weight (g)	0.024 ± 0.001	0.032 ± 0.002	0.023 ± 0.002	0.022 ± 0.001	0.032 ± 0.002	0.029 ± 0.002	0.027 ± 0.001	NS
Sixth leaf weight (g)	0.036 ± 0.002	0.036 ± 0.002	0.037 ± 0.002	0.027 ± 0.001	0.038 ± 0.002	0.027 ± 0.001	0.033 ± 0.002	NS
Seventh leaf weight (g)	0.009 ± 0.0008	0.009 ± 0.0007	0.005 ± 0.0002	0.006 ± 0.0002	0.005 ± 0.0003	0.005 ± 0.0005	0.003 ± 0.0006	NS

Mean ± standard error and significance of ANOVA are presented for each variety

*NS* non-significant

* p < 0.05; ** p < 0.001

**Table 3:** Growth parameters obtained by imaging and ANOVA for seven rice genotypes at 14 days after sowing

Traits	LB-46	GM-217	AC38399	ARC10656	Vandana	Sabita	Varshadhan	ANOVA
First leaf length (mm)	52.47 ± 5.79	39.33 ± 2.62	47.86 ± 5.29	48.90 ± 2.22	55.69 ± 1.89	61.88 ± 3.97	44.45 ± 4.16	*
Second leaf length (mm)	131.17 ± 16.29	125.18 ± 7.25	143.59 ± 6.96	120.87 ± 8.15	147.78 ± 3.62	112.42 ± 6.39	118.97 ± 4.30	NS
Third leaf length (mm)	178.21 ± 10.2	202.07 ± 4.17	173.02 ± 10.95	158.66 ± 9.32	203.80 ± 4.40	118.24 ± 17.82	158.06 ± 8.61	**
First leaf width (mm)	2.39 ± 0.48	1.83 ± 0.15	2.40 ± 0.24	2.02 ± 0.14	2.83 ± 0.11	2.54 ± 0.23	2.43 ± 0.12	NS
Second leaf width (mm)	2.38 ± 0.21	2.53 ± 0.13	2.80 ± 0.25	2.51 ± 0.14	2.78 ± 0.11	2.96 ± 0.30	2.68 ± 0.07	NS
Third leaf width (mm)	2.96 ± 0.23	2.20 ± 0.13	3.12 ± 0.43	2.4 ± 0.35	3.81 ± 0.12	2.61 ± 0.25	3.61 ± 0.27	**
First leaf area (mm^2^)	97.38 ± 20.53	81.10 ± 11.53	107.57 ± 17.43	115.90 ± 13.72	114.28 ± 7.06	107.52 ± 7.72	80.29 ± 12.35	NS
Second leaf area (mm^2^)	242.33 ± 38.73	257.90 ± 17.78	305.80 ± 31.68	214.34 ± 18.34	276.31 ± 15.97	217.15 ± 31.27	225.43 ± 23.62	NS
Third leaf area (mm^2^)	381.01 ± 45.32	352.48 ± 10.54	374.25 ± 57.70	268.87 ± 40.13	511.13 ± 30.33	221.04 ± 46.28	351.85 ± 54.56	**
Stem area (mm^2^)	185.89 ± 54	402.86 ± 30.15	176.98 ± 20.04	199.65 ± 25.43	216.80 ± 15.28	143.79 ± 13.42	171.66 ± 12.39	**
Eccentricity	205.37 ± 22.72	287.20 ± 23.04	148.08 ± 31.8	164.477 ± 17.45	241.80 ± 17.06	110.20 ± 12.64	160.49 ± 18.61	**
Convex hull (mm^2^)	9029.58 ± 3243	24191.40 ± 3913.40	4624.74 ± 1784	6657.24 ± 2597	17011.46 ± 3955	2635.28 ± 679.39	5376.21 ± 1912	**
Caliper length (mm)	311.49 ± 48.16	403.99 ± 25.76	227.75 ± 49	207.89 ± 21.76	415.39 ± 22.75	162.58 ± 19.40	286.71 ± 34.94	**
WPAs (mm^2^)	906.62 ± 89	1264.52 ± 98	964.61 ± 98.64	798.76 ± 44.57	1118.54 ± 49.51	689.49 ± 68.34	829.22 ± 75.42	**
WPA_i_ (mm^2^)	1095.45 ± 178	1498.38 ± 124	726.61 ± 143	593.90 ± 61.76	1384.60 ± 86.28	514.22 ± 57.06	857.27 ± 138.47	**
Top view area (mm^2^)	3061.90 ± 642.51	3222.871 ± 848.06	1637.00 ± 591	1359.82 ± 337	1057.60 ± 284	1057.60 ± 284.62	1539.68 ± 422	**
Compactness	0.34 ± 0.04	0.13 ± 0.01	0.35 ± 0.03	0.20 ± 0.02	0.06 ± 0.01	0.40 ± 0.05	0.29 ± 0.02	**

Mean ± standard error and significance of ANOVA are presented for each variety

*NS* non-significant

* p < 0.05; ** p < 0.001

**Table 4:** Growth parameters obtained by imaging and ANOVA for seven rice genotypes at 28 days after sowing

Traits	LB-46	GM-217	AC38399	ARC10656	Vandana	Sabita	Varshadhan	ANOVA
First leaf length (mm)	66.32 ± 2.76	63.20 ± 13.26	48.21 ± 3.08	67.36 ± 16.78	72.14 ± 18.87	101.26 ± 19.02	48.21 ± 3.08	NS
Second leaf length (mm)	111.94 ± 20.43	147.49 ± 16.44	130.81 ± 4.61	128.46 ± 19.29	173.22 ± 20.30	126.56 ± 29.26	130.81 ± 4.61	NS
Third leaf length (mm)	151.18 ± 20.03	193.23 ± 22.2	180.63 ± 4.42	177.35 ± 15.42	229.31 ± 21.69	174.90 ± 31.52	180.63 ± 4.42	NS
Fourth leaf length (mm)	198.58 ± 15.05	229.58 ± 21.52	210.45 ± 4.95	228.71 ± 25.32	271.79 ± 26.66	201.28 ± 27.44	210.45 ± 4.95	NS
Fifth leaf length (mm)	243.21 ± 21.18	229.89 ± 37.97	230.32 ± 11.47	234.56 ± 39.73	205.24 ± 44.85	180.11 ± 27.27	230.32 ± 13.31	NS
Sixth leaf length (mm)	229.48 ± 44.81	168.76 ± 74.47	229.27 ± 41.11	190.79 ± 92.98	184.21 ± 81.17	255.59 ± 21.16	229.27 ± 41.11	NS
Seventh leaf length (mm)	201.18 ± 39.41	582.00 ± 82.14	74.13 ± 14.64	85.64 ± 17.24	83.14 ± 19.62	228.57 ± 24.51	74.13 ± 4.91	NS
First leaf width (mm)	2.16 ± 0.34	2.18 ± 0.28	2.89 ± 0.37	2.11 ± 0.33	3.50 ± 0.20	3.60 ± 0.21	2.89 ± 0.37	**
Second leaf width (mm)	2.62 ± 0.24	2.66 ± 0.41	2.77 ± 0.16	2.49 ± 0.27	3.34 ± 0.32	3.74 ± 0.23	2.77 ± 0.16	*
Third leaf width (mm)	3.19 ± 0.42	3.03 ± 0.52	3.68 ± 0.16	2.88 ± 0.32	4.39 ± 0.36	3.96 ± 0.37	3.68 ± 0.16	*
Fourth leaf width (mm)	4.36 ± 0.50	3.42 ± 0.24	4.78 ± 0.34	3.36 ± 0.08	4.57 ± 0.44	4.22 ± 0.36	4.78 ± 0.34	**
Fifth leaf width (mm)	4.61 ± 0.91	14.25 ± 10.88	5.25 ± 0.26	2.79 ± 0.39	3.34 ± 0.61	4.14 ± 0.61	5.25 ± 0.26	NS
Sixth leaf width (mm)	4.34 ± 0.90	3.03 ± 0.72	4.33 ± 0.87	2.63 ± 0.42	3.17 ± 0.69	4.16 ± 0.14	4.33 ± 0.87	NS
Seventh leaf width (mm)	5.31 ± 0.86	5.42 ± 0.92	0.93 ± 0.08	0.98 ± 0.10	0.94 ± 0.08	4.02 ± 0.17	0.93 ± 0.11	NS
First leaf area (mm^2^)	115.62 ± 15.13	99.29 ± 23.83	96.96 ± 14.69	118.96 ± 41.85	166.62 ± 37.83	253.11 ± 56.69	96.96 ± 14.69	*
Second leaf area (mm^2^)	225.30 ± 46.01	241.81 ± 56.29	237.80 ± 18.04	250.32 ± 50.13	413.61 ± 86.04	339.22 ± 85.60	237.81 ± 18.04	NS
Third leaf area (mm^2^)	351.65 ± 74.75	385.22 ± 91.89	397.66 ± 12.85	361.76 ± 59.46	690.66 ± 142.21	482.88 ± 110.97	397.66 ± 12.85	NS
Fourth leaf area (mm^2^)	604.80 ± 115.2	559.96 ± 65.80	583.40 ± 24.37	492.26 ± 62.38	845.77 ± 145.52	598.48 ± 86.07	583.40 ± 24.37	NS
Fifth leaf area (mm^2^)	873.96 ± 151.6	613.48 ± 85.58	734.45 ± 90.82	485.48 ± 118.77	495.43 ± 148.94	460.41 ± 137.46	734.46 ± 90.82	NS
Sixth leaf area (mm^2^)	881.14 ± 258.6	546.32 ± 269.52	743.25 ± 176.97	372.59 ± 232.86	378.47 ± 214.36	790.56 ± 65.25	743.25 ± 176.97	NS
Seventh leaf area (mm^2^)	811.20 ± 196.4	998.36 ± 257.31	52.21 ± 8.54	65.92 ± 10.35	62.34 ± 14.68	573.51 ± 55.74	52.21 ± 7.24	NS
Stem area (mm^2^)	647.09 ± 84.22	856.64 ± 60.32	650.17 ± 54.88	630.66 ± 30.95	725.42 ± 85.67	726.68 ± 27.03	650.17 ± 54.88	NS
Eccentricity	285.21 ± 33.9	311.64 ± 24.15	234.53 ± 24.43	196.20 ± 20.69	196.91 ± 15.77	169.86 ± 24.42	260.95 ± 33.49	**
Convex hull (mm^2^)	80580.53 ± 15441	58032.17 ± 14316	21874.73 ± 6181	20969.94 ± 4023	16674.40 ± 2546	24296.94 ± 6958	35618.86 ± 13802.31	**
Caliper length (mm)	510.05 ± 66.91	457.71 ± 74.49	374.78 ± 29.91	305.70 ± 35.86	327.26 ± 29.27	302.32 ± 49.05	451.99 ± 58.82	*
WPAs (mm^2^)	4522.00 ± 503	3585.16 ± 406.04	3510.24 ± 406.96	2488.48 ± 97.75	3337.50 ± 293.75	3107.58 ± 74.03	3454.15 ± 248.33	**
WPA_i_ (mm^2^)	4068.50 ± 938	2215.22 ± 580.52	2032.65 ± 310	1109.57 ± 50.02	1456.73 ± 150.38	1744.54 ± 149.50	2483.14 ± 399.20	**
Top view area (mm^2^)	8940.64 ± 3461	3052.59 ± 377	4107.37 ± 1301	2092.91 ± 421	2419.07 ± 537.93	3578.04 ± 894.76	5536.39 ± 1623	**
Compactness	0.11 ± 0.08	0.05 ± 0.01	0.19 ± 0.06	0.10 ± 0.06	0.15 ± 0.07	0.15 ± 0.08	0.16 ± 0.07	**

Mean ± standard error and significance of ANOVA are presented for each variety

*NS* non-significant

* p < 0.05; ** p < 0.001

### Comparison between destructive sampling and automated image phenotyping (WPA_i_) for seedling vigor

**Fig. 1:**
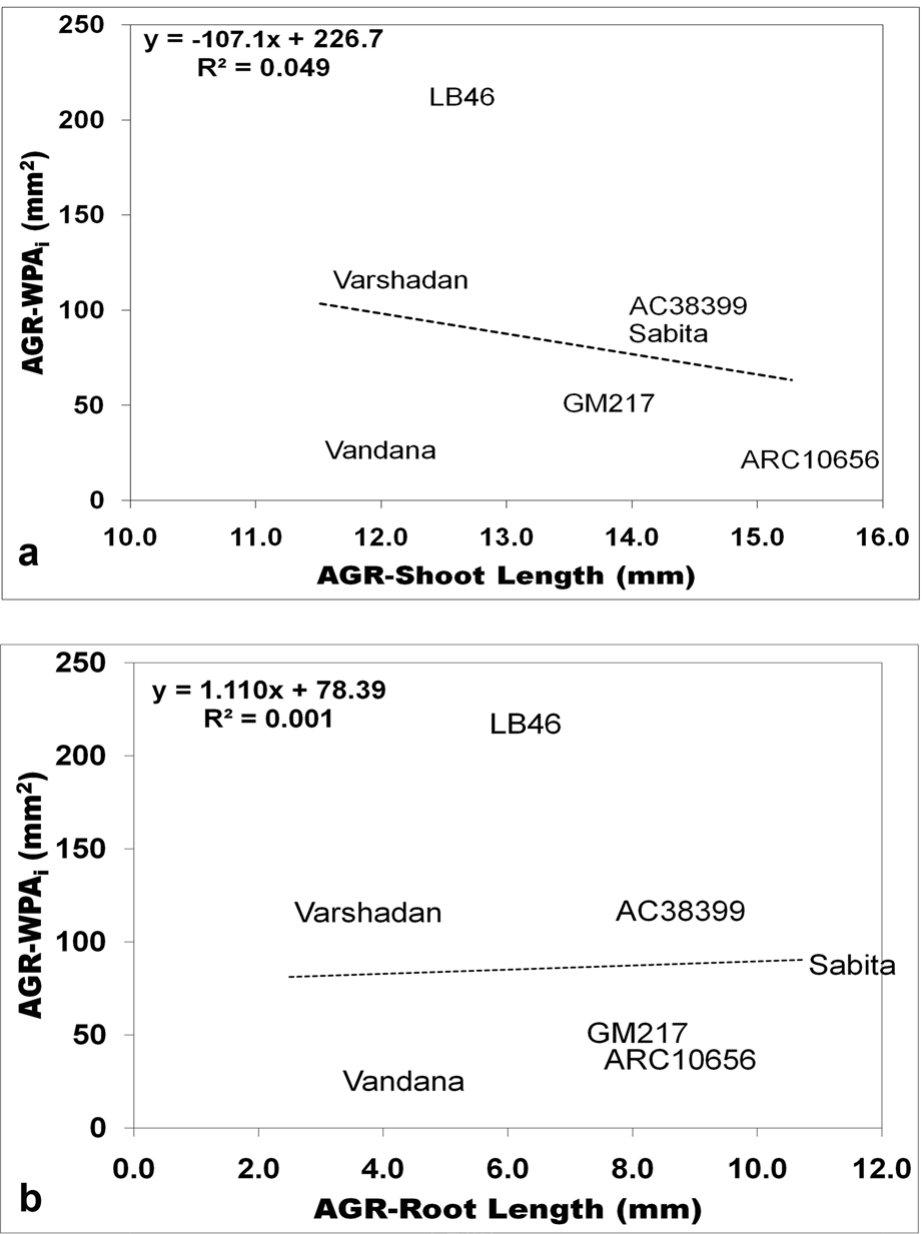
Result of linear regression analysis showing no correlation between the absolute growth rate (AGR) of morphological traits (shoot and root length) and AGR-WPA_i_. **a** AGR-shoot length vs AGR-WPA_i_, **b** AGR-root length vs AGR-WPA_i_. The line indicates the fitted results representing the relationship between AGR of morphological traits and AGR-WPA_i_, *AGR* absolute growth rate

**Fig. 2:**
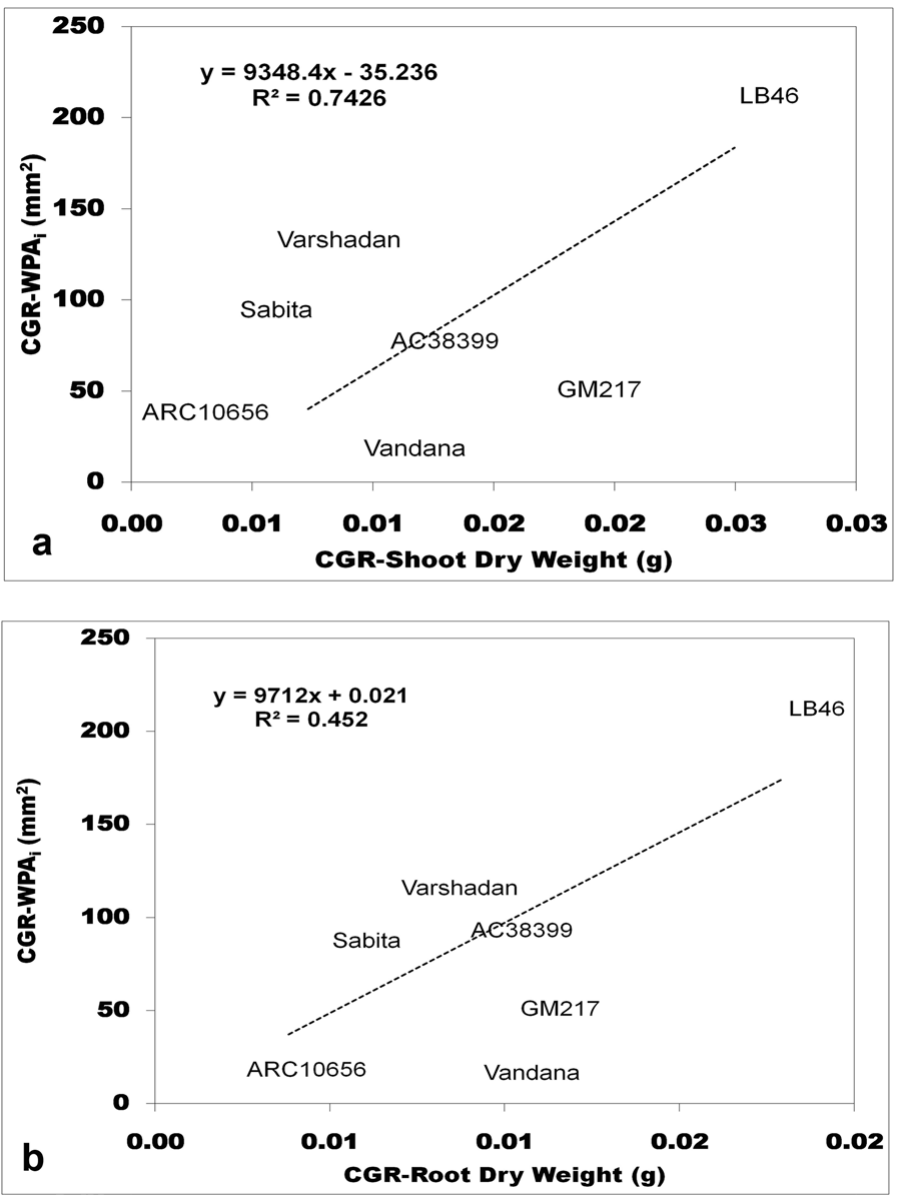
Result of linear regression analysis showing a positive correlation between crop growth rate (CGR) of morphological traits (shoot and root dry weight) and CGR-WPA_i_. **a** CGR-shoot dry weight vs CGR-WPA_i_, **b** CGR-root dry weight vs CGR-WPA_i_. The line indicates the fitted results representing the relationship between CGR of morphological traits and CGR-WPA_i_, *CGR* crop growth rate

**Fig. 3:**
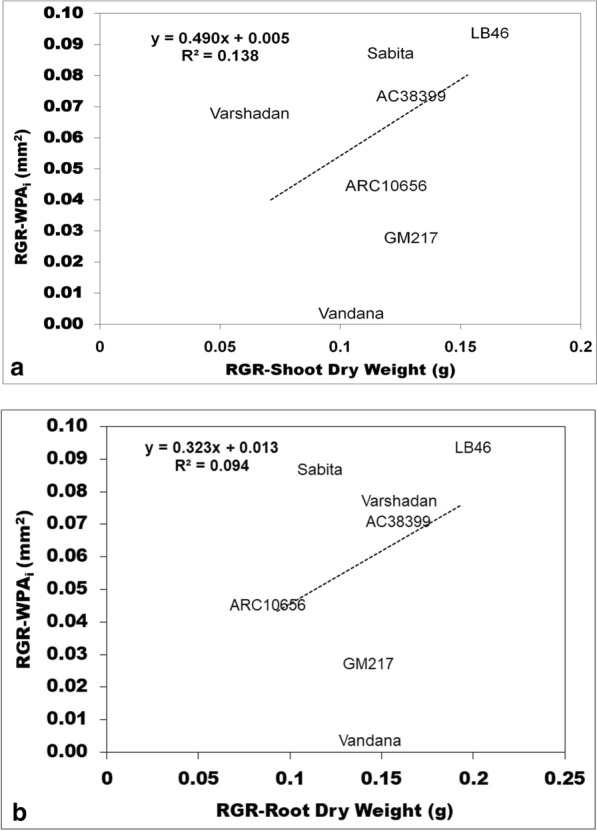
Result of linear regression analysis showing a weak correlation between relative growth rate (RGR) of morphological traits (shoot and root dry weight) and RGR-WPA_i_. **a** RGR-shoot dry weight vs RGR-WPA_i_, **b** RGR-root dry weight vs RGR-WPA_i_. The line indicates the fitted results representing the relationship between RGR of morphological traits and RGR-WPA_i_, *RGR* relative growth rate

**Fig. 4:**
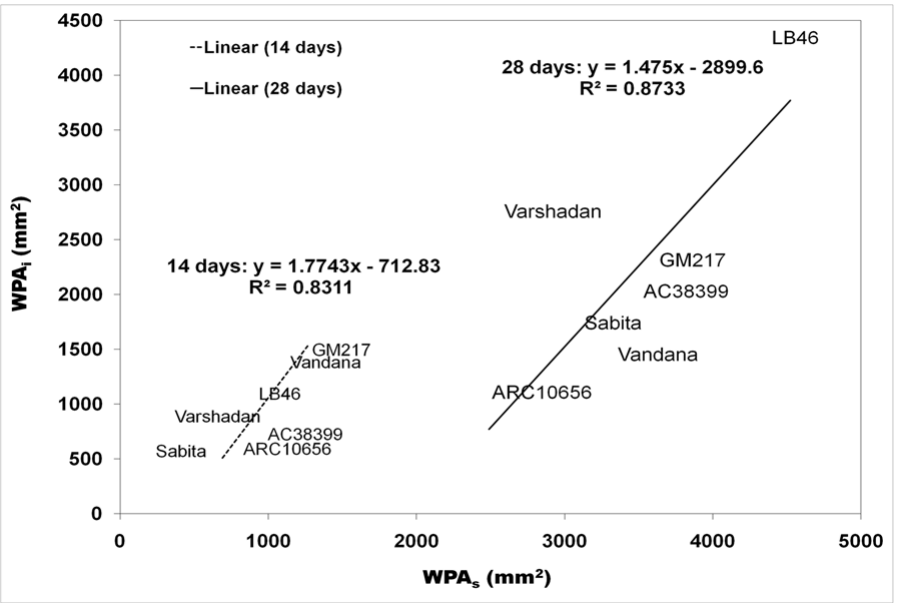
Result of linear regression analysis showing a positive correlation between whole-plant area by destructive-flatbed scanner (WPA_s_) and whole-plant area by non-destructive imaging (WPA_i_) at 14 and 28 days after sowing. The line indicates the fitted results representing the relationship between WPA_s_ and WPA_i_

Seedling vigor was generally predicted conventionally through growth analysis. Growth analysis was calculated as the absolute growth rate (AGR), crop growth rate (CGR), and relative growth rate (RGR), which are destructive methods (explained in detail under the Methods section (Method-2 & -3). All three of these growth parameters at 14 and 28 DAS were plotted against WPA_i_ (non-destructive method) using regression curves to find out the association and contribution in variation for WPA_i_. In the present experiment, WPA_i_ is considered a principal parameter from image-based phenotyping, since WPA is the target trait measured through automated image analysis. AGR was calculated from shoot and root length, while CGR and RGR were calculated based on the dry weight of shoots with tillers and roots. The AGR for shoot length and root length plotted against the AGR of WPA_i_ exhibited no relationship with the AGR of WPA_i_ with nearly zero regression (≤ 0.04) (Fig. 1a, b). On the other hand, CGR of shoot dry weight with tillers and root dry weight exhibited strong and positive correlation with CGR of WPA_i_ (Fig. 2a, b). The CGR of shoot dry weight with tillers explained 74.26% of the variation and the CGR of root dry weight explained 45.20% of the variation of CGR-WPA_i_. Similarly, RGR of shoot dry weight with tillers and root dry weight showed a positive relationship with the RGR of WPA_i_ (Fig. 3a, b). However, only the RGR of shoot dry weight had a positive correlation and explained 13.80% of the variation of the RGR of WPA_i_. Thus, the CGR of shoot and root dry weight and RGR of shoot dry weight had a positive relationship with CGR of WPA_i_ and RGR of WPA_i_, respectively. Another method of destructive sampling was WPAs, which was a more precise method than the phenotypic measurement for WPA_i_ estimation. Both WPA_i_ and WPAs were plotted on the graph, where WPA_s_ was plotted on the X-axis against WPA_i_ on the Y-axis as a dependent variable. The correlation of WPAs at both 14 and 28 DAS was strong and positive with WPA_i_. The regression showed that WPAs explained 83.11% of the variation at 14 DAS and 87.33% of the variation at 28 DAS of WPA_i_ (Fig. 4). This was the strongest relationship among all the traits observed.

### Relationship between WPA_i_ and other seedling traits

The relationship of seedling phenotypic traits, individual leaf traits, stem area, and WPA_s_ with WPA_i_ was calculated to understand the correlation and percentage of variation contributed by each trait (morphological traits and geometric traits) toward WPA_i_. As WPA_i_ was used as a trait in the identification of early seedling vigor in plants, it was considered as a primary parameter in the image-based phenotyping method.

#### Relationship of seedling phenotypic traits and geometric traits with WPA_i_

Both positive and negative correlation was observed between seedling traits and WPA_i_. At 28 DAS, WPA_i_ had exhibited a strong positive relationship with morphological traits such as the number of leaves (r = 0.95, p < 0.01), shoot and root dry weight, and tiller number (Table 5). In general, no strong negative association was observed other than with some specific leaves at 28 DAS (presented in the following section). Similarly, geometric traits that related to the size of the plant such as caliper length (r = 0.88, p < 0.05), convex hull, and top view area had a strong and positive correlation with WPA_i_ at 28 DAS. Parallel to the data observed at 28 DAS, traits observed at 14 DAS also exhibited a similar trend of relationship (Table 5). The contribution of variation by shoot length explained 90.4% of the WPA_i_ variation at 14 DAS, while it had a negative contribution of 3.6% at 28 DAS. At 28 DAS, 91.20% of the variation in WPA_i_ was explained by the number of leaves (Table 5).

**Table 5:** Correlation and regression coefficient between traits observed (phenotypic and geometric) and WPA_i_ at 14 and 28 DAS

Traits	Regression coefficient (%)		Correlation coefficient (r)	
	14 days	28 days	14 days	28 days
Shoot length	90.40	− 3.60	0.951**	− 0.190 ^ns^
Root length	− 2.30	− 11.80	− 0.154 ^ns^	− 0.344 ^ns^
Shoot dry weight	9.60	86.30	0.311 ^ns^	0.929**
Root dry weight	34.11	74.40	0.584 ^ns^	0.863*
Seed weight with mesocotyl	− 12.00	–	0.732 ^ns^	–
Mesocotyl length	− 10.00	–	0.496 ^ns^	–
Stem weight	–	82.30	–	0.920**
Tiller number	–	79.80	–	0.970**
Stem thickness	17.70	27.30	0.422 ^ns^	0.526 ^ns^
Leaf number/plant	30.30	91.20	0.551 ^ns^	0.955**
First leaf weight	− 1.20	− 24.10	0.241 ^ns^	− 0.491 ^ns^
Second leaf weight	40.00	9.60	0.645 ^ns^	0.318 ^ns^
Third leaf weight	54.90	− 0.01	0.661 ^ns^	− 0.001 ^ns^
Fourth leaf weight	–	–	–	− 0.503 ^ns^
Fifth leaf weight	–	–	–	− 0.188 ^ns^
Six leaf weight	–	17.50	–	0.419 ^ns^
Seventh leaf weight	–	15.00	–	0.192 ^ns^
First leaf length	18.52	5.19	− 0.398 ^ns^	− 0.227 ^ns^
Second leaf length	21.19	29.26	0.470 ^ns^	− 0.538 ^ns^
Third leaf length	77.64	38.41	0.892**	− 0.614 ^ns^
Fourth leaf length	–	27.67	–	− 0.550 ^ns^
Fifth leaf length	–	21.77	–	0.467 ^ns^
Sixth leaf length	–	6.80	–	0.374 ^ns^
Seventh leaf length	–	5.09	–	0.287 ^ns^
First leaf width	0.73	15.89	− 0.074 ^ns^	− 0.364 ^ns^
Second leaf width	− 6.55	9.74	− 0.367 ^ns^	− 0.303 ^ns^
Third leaf width	2.33	8.42	0.150 ^ns^	− 0.242 ^ns^
Fourth leaf width	–	7.99	–	0.290 ^ns^
Fifth leaf width	–	2.54	–	0.163 ^ns^
Sixth leaf width	–	24.23	–	0.526 ^ns^
Seventh leaf width	–	35.33	–	0.634 ^ns^
First leaf area	14.26	9.30	− 0.390 ^ns^	− 0.305 ^ns^
Second leaf area	18.02	24.42	0.400 ^ns^	− 0.494 ^ns^
Third leaf area	51.29	18.06	0.730 ^ns^	− 0.425 ^ns^
Fourth leaf area	–	00.75	–	− 0.087 ^ns^
Fifth leaf area	–	77.79	–	0.882**
Sixth leaf area	–	53.25	–	0.644 ^ns^
Seventh leaf area	–	14.65	–	0.395 ^ns^
Stem area	61.33	1.59	0.840**	− 0.126 ^ns^
Eccentricity	− 27.30	45.80	0.960**	0.677 ^ns^
Convex hull	− 25.20	79.20	0.927**	0.890**
Caliper length	− 48.30	77.60	0.984**	0.881**
Top view area	− 1.90	93.20	0.562 ^ns^	0.965**
Compactness	17.60	− 1.00	0.725 ^ns^	− 0.101 ^ns^

*NS* non-significant

* p < 0.05; ** p < 0.001

Geometric traits such as caliper length, eccentricity, convex hull, and top view area explained 48.3%, 27.3%, 25.2%, and 1.9% of the variation, respectively, at 14 DAS in a negative direction. Conversely, at 28 DAS, they explained the variation positively (Table 5). Overall, seedling phenotypic traits and geometric traits were highly correlated with WPA_i_ and thus can be predictable using linear regression.

#### Relationship of the different leaves with WPA_i_

The relationship between WPA_i_ and individual leaf length, width, and area at 14 and 28 DAS was estimated using a linear regression curve (Table 5). There were three leaves per plant at 14 DAS and seven leaves at 28 DAS in all the genotypes. Some genotypes produced a fourth leaf at 14 DAS and some produced an eighth leaf at 28 DAS. Therefore, the fourth leaf (at 14 DAS) and eighth leaf (at 28 DAS) were excluded from the analysis. Both positive and negative regression were observed between WPA_i_ and individual leaf length. Among all the leaves, third leaf length had a positive (0.89, p < 0.01) correlation with WPA_i_, which explained 77.64% of the WPA_i_ variation at 14 DAS. Similarly, length of the fifth-seventh leaf at 28 DAS had a positive association and explained > 40% of the variation (Table 5).

With reference to leaf width, no strong relationship between WPA_i_ and leaf width was observed at both 14 and 28 DAS, although the highest variation was explained by seventh leaf width (35.33%) at 28 DAS (Table 5). In the case of leaf area, the second-third leaves had a positive relationship with WPA_i_ at 14 DAS and explained 51.29% of the WPA_i_ variation. For 28 DAS, the fifth-seventh leaves had a positive relationship with WPA_i_, which was similar to the results obtained for leaf length. However, the leaf area of the fifth and sixth leaves had a strong correlation, which explained the extent of variation (77.79% and 53.25%, respectively) of WPA_i_. For leaf weight, all leaves had a positive correlation (Table 5). Among them, the second and third leaves of 14-day-old seedlings explained variation of more than 40%. On the other hand, the leaf weight of 28-day-old seedlings at different levels had a weak correlation with WPA_i_. Of these, the sixth and seventh leaves explained variation of > 15%.

### Relationship of stem area with WPA_i_ and stage-specific traits with WPA_i_

The greenness in the stem also helps in photosynthesis, which contributes to the overall growth of seedlings. Hence, stem area was also measured to find out the relationship with WPA_i_. The relationship between stem area and WPA_i_ was positive (0.84, p < 0.01) at 14 DAS and negative (0.12^ns^) at 28 DAS. At 14 DAS, a strong relationship was observed, which explained 61.33% of the variation, while at 28 DAS the correlation was negative (1.59%) and very weak. Some of the unique traits in correlation to seedlings were also measured, which were seedling age-specific. Traits such as seed weight with mesocotyl, seed weight, and mesocotyl length were measured at 14 DAS and bulk eighth leaf (terminal) weight, stem weight, and tiller number were measured at 28 DAS. The traits that were measured at 14 DAS were not available to measure at 28 DAS and vice versa. The traits seed weight with mesocotyl, seed weight, and mesocotyl length were negative and had a weak correlation with WPA_i_ at 14 DAS, whereas bulk eighth leaf weight, stem weight, and tiller number showed a positive relationship with WPA_i_ at 28 DAS. Single stem weight and tiller number had a strong relationship with WPA_i_ and explained 82.30% and 79.80% of the variation of WPA_i_, respectively.

### Relationship between morphological traits and geometric traits

Understanding the trait association between morphological and geometric traits observed by image analysis would be helpful in the identification of surrogate traits in the absence of an automated non-destructive imaging system. At 28 DAS, number of leaves per plant expressed a strong positive association with top view area (0.89, p < 0.01), WPA_i_ (0.95), caliper length (0.91, p < 0.01), convex hull (0.83), root dry weight (0.81, p < 0.05), shoot dry weight (0.93, p < 0.01), tiller number (0.97, p < 0.01), and stem weight (0.88, p < 0.01). The RGR-related trait compactness derived from the differences in top view area and convex hull exhibited a strong positive association with leaf width irrespective of all levels and a strong negative association with shoot length, eccentricity, convex hull, and caliper length on both dates of observation. Further, it has a weak negative association with leaf number (− 0.20 at 14 DAS and − 0.28 at 28 DAS). The parameter convex hull displays the degree of leaves spreading that helps to cover the ground. The number of leaves showed a strong positive association with convex hull on both observation dates (0.55 at 14 DAS and 0.83 at 28 DAS) and a negative association with leaf width at all levels.

### Identification of genotypes with high seedling vigor

Generally, to identify genotypes with high seedling vigor, AGR, CGR, and RGR were used. Among these, CGR was commonly used to find vigorous genotypes. The CGR, RGR, and AGR of genotypes were compared to the WPA_i_ of the respective growth rates. The CGR of shoot weight and root weight, AGR of shoot length and root length, and RGR of root dry weight and shoot dry weight were plotted against the CGR of WPA_i_, AGR of WPA_i_, and RGR of WPA_i_, respectively. The highest growth rate was observed in LB-46 (CGR: 0.02 (shoot dry weight) and 0.01 (root dry weight); RGR: 0.15 (shoot dry weight) and 0.19 (root dry weight)), considered as a genotype with high seedling vigor, followed by GM-217 and Varshadhan, based on the destructive method (CGR and RGR) for shoot and root dry weight. Meanwhile, AGR showed that ARC10656 and AC38399 were superior to other genotypes.

Image analysis by the non-destructive way has identified LB-46 (CGR of WPA_i_: 212.36; RGR of WPA_i_: 0.09) as a genotype with high seedling vigor, followed by Varshadhan. In both methods, LB-46 is common. Thus, WPA_i_ is certainly an alternative to the destructive method (Fig. 4). On the basis of imaging and scanning methods (WPA_i_ and WPA_s_) at 28 DAS, the highest WPA_i_ was gained in order as LB-46 (4068 mm^2^), Varshadhan, and GM-217, while LB-46, GM-217, AC38399, and Varshadhan were judged as top genotypes by the WPA_s_ method (Table 4) (Fig. 4). Overall at 14 DAS, both WPA_i_ and WPA_s_ identified GM-217 (1498 mm^2^ (WPA_i_)/1264 mm^2^ (WPA_s_)) and Vandana as top contenders, followed by LB-46 in WPA_i_ and AC38399 in WPA_s_ as the next best genotypes (Table 3).

### Grouping pattern of genotypes and association between variables

**Fig. 5:**
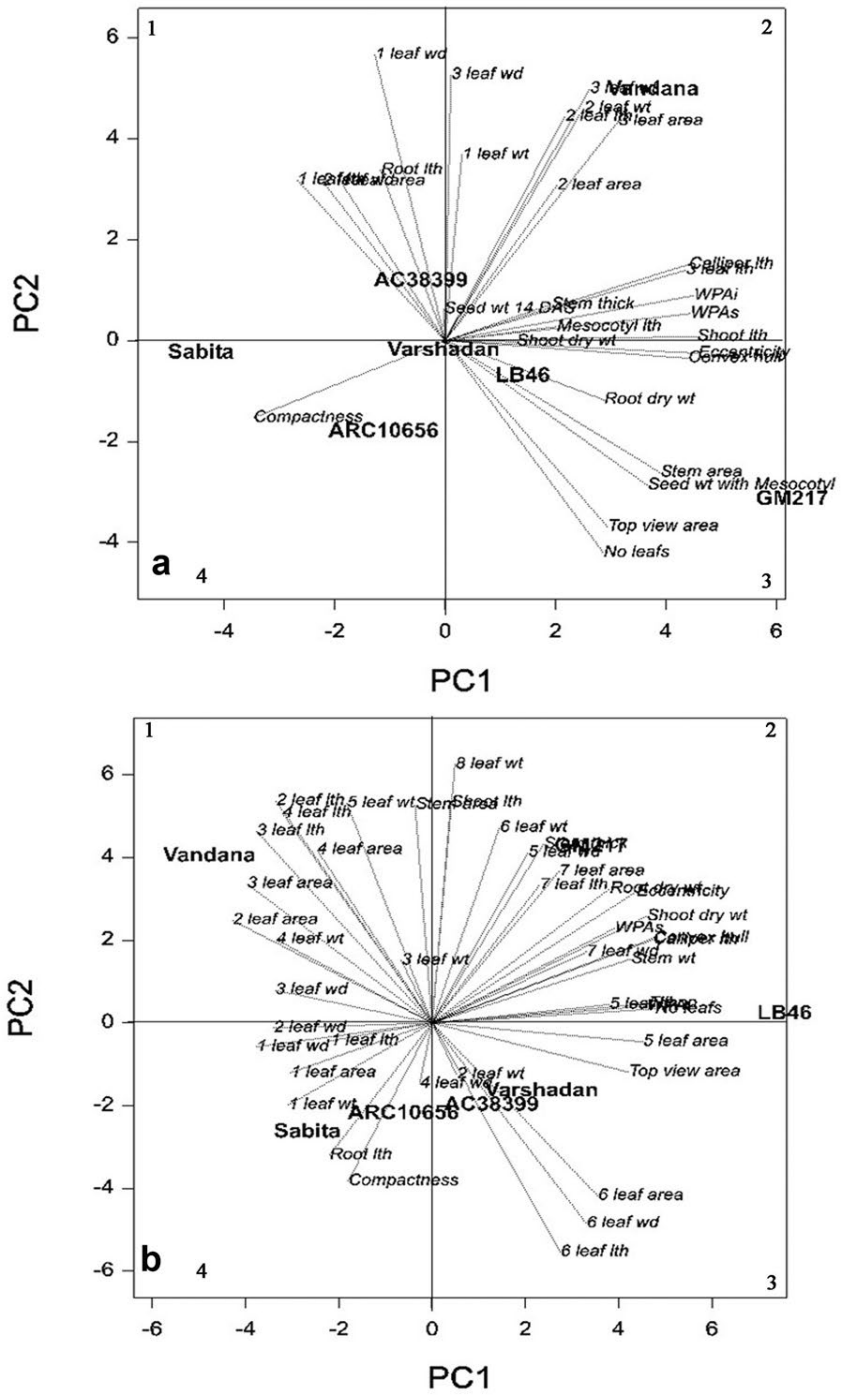
Spatial distribution of genotypes based on seedling vigor traits for the first two principal components. **a** Distribution of genotypes based on 29 seedling vigor traits at 14 DAS, **b** Distribution pattern of genotypes based on 44 seedling vigor traits at 28 DAS

Principal component analysis (PCA) was employed for 29 traits observed at 14 DAS, for which it has explained 98.99% of the variation by PC1 and 0.96% by PC2 (Fig. 5a). On the basis of magnitudes of loadings/eigenvalues, nine highly variable traits (first and third leaf area, caliper length, convex hull, eccentricity, stem area, top view area, WPAi, WPAs) were identified. Similarly, PCA was performed for 44 traits at 28 DAS, which governs 99.49% of the variation on the PC1 axis and 0.47% on the PC2 axis (Fig. 5b). Out of 44 traits on the basis of magnitude of PCA, fifth, sixth, and seventh leaf area; sixth and seventh leaf length; convex hull; stem area; top view area; WPAi; and WPAs were identified as highly variable traits. Among them, convex hull, stem area, top view area, WPAi, and WPAs were highly variable common traits between the two dates of observation.

The cultivar-by-trait biplots (Fig. 5a, b) were analyzed for both 14 and 28 DAS. They showed a strong relationship between WPAi and WPAs at both 14 and 28 DAS. On both dates, biplot classifies the traits into two groups, based on their association with whole-plant area (WPAi and WPAs). At 14 DAS, traits such as width, area, and dry weight of first, second, and third leaves; leaf length of first and second leaves; and root length were clustered together. Conversely, traits that related to the image that contributes directly to WPA such as shoot length, caliper length, eccentricity, convex hull, third leaf length, stem thickness, shoot dry weight, stem area, top view area, and leaf number were grouped together. The vector of compactness stayed away from both groups. A similar trend was also observed at 28 DAS. Traits such as leaf length, width, weight, and area of 1–4; leaf dry weight of 5–8; stem area; root length; and compactness were grouped together. On the other hand, geometric and morphological traits that contributed to WPA such as shoot length; seventh leaf length, width, and weight; shoot length; shoot dry weight; stem weight; caliper length; convex hull; eccentricity; leaf length, width, and area of fifth and sixth leaf; top view area; and leaf number were clustered together. As the seedling grows, the association of stem area and compactness was clustered with traits that had a minimum role in estimating WPA_i_ and WPA_s_. In agreement with the preceding section, genotypes LB-46, GM-217, and Varshadhan exhibited the highest magnitude toward geometric traits on both dates of observation.

## Discussion

### Rationale of early seedling vigor trait in rice

As DSR saves water and labor [1, 22], it is becoming popular across all fronts of rice ecosystems by covering 29 million ha of the rice area in Asia (21% of the total rice area) [23]. Good crop establishment is a major challenge in the DSR system. Germinating seeds and/or seedlings under DSR are exposed directly to an array of stresses [1, 7]. Thus, strong and early seedling vigor is an imperative breeding trait for DSR varieties. Rice cultivars with high early seedling vigor decrease crop yield loss due to weeds (16% yield loss), water [24], and nutrient by exceeding the growth of weeds [25]. In addition, they help to achieve rapid and uniform emergence from the field. Genetic improvement for early vigor has been practiced in rice for better crop establishment. Caton et al. [26] reported that early vigor was a highly repeatable trait among rice cultivars. Further, 87% grain yield variation in rice was accounted for by vegetative vigor (2 weeks after sowing) in a comparison between weed and weed-free field conditions [27]. Therefore, the identification and use of suitable donors with relevant traits associated with early seedling vigor and variability available in genotypes are essential. This helps in selecting traits and designing plants for early seedling vigor. However, the introgression of seedling vigor into any recipient parent or selection of lines with seedling vigor in segregating generations could not be achieved with destructive sampling. This limits breeding for seedling vigor traits. The absence of a non-destructive method to estimate seedling vigor hampers the whole experiment and creates hurdles in exploiting early seedling vigor in rice. Thus, a robust automated non-destructive image phenotyping technique will help to overcome these limitations in the area of early seedling vigor. Seedling vigor has several component traits: biomass accumulation, canopy coverage, plant height, etc. These components are traditionally recorded visually and in a destructive way [28, 29]. To establish a relation of those traits with seedling vigor and to judge the robustness of WPA_i_, an automated non-destructive phenotyping technique in rice was developed and the method established with genetically diverse rice genotypes. Phenotyping using RGB imaging has been designed for field crops, often for abiotic stresses (drought, salinity, and cold) [15, 30, 31], but, surprisingly, rare attempts were made to screen traits such as early seedling vigor.

### The necessity of automated image-based phenotyping for seedling vigor

Early seedling vigor is a polygenic trait, and it requires measurements of phenotypic data of component traits for genetic dissection into smaller manageable and measurable components [32]. Conventionally, early seedling vigor assessment involves manual visual scoring, leaf area measurement, shoot biomass measurement, etc. [28, 33]. Manual methods are labor-intensive, in particular, the measurements are prone to human error, manual data management, and data keeping, and may not be suitable for handling a large number of samples. In rice, 2-week-old seedlings are small and delicate, and often lead to error. Therefore, robust and automated phenotyping platforms that can capture high-quality and reliable phenotypic data would be error-free and straightforward to handle. Image-based phenotyping offers several advantages over destructive methods, in which digital color images are used to quantify phenotype [13, 14, 34]. In our experiment, we established a phenotypic platform with available resources. It saves nearly 80% of the time (660 s were required per sample of five plants with four persons for observing data by destructive sampling method vis-à-vis two persons with 152 s of proposed imaging protocol) and ~ 50% on the cost of labor. A population developed for early seedling vigor would segregate for the early seedling vigor component traits and differ at the genic level. Therefore, destructive sampling for early seedling vigor populations might lead to a loss in variation and deviation from a normal probability distribution and Hardy–Weinberg law with biased results. Therefore, image-based phenotyping would overcome those constraints to achieve precise phenotyping with better reproducibility.

### Destructive versus non-destructive method of phenotyping

**Fig. 6:**
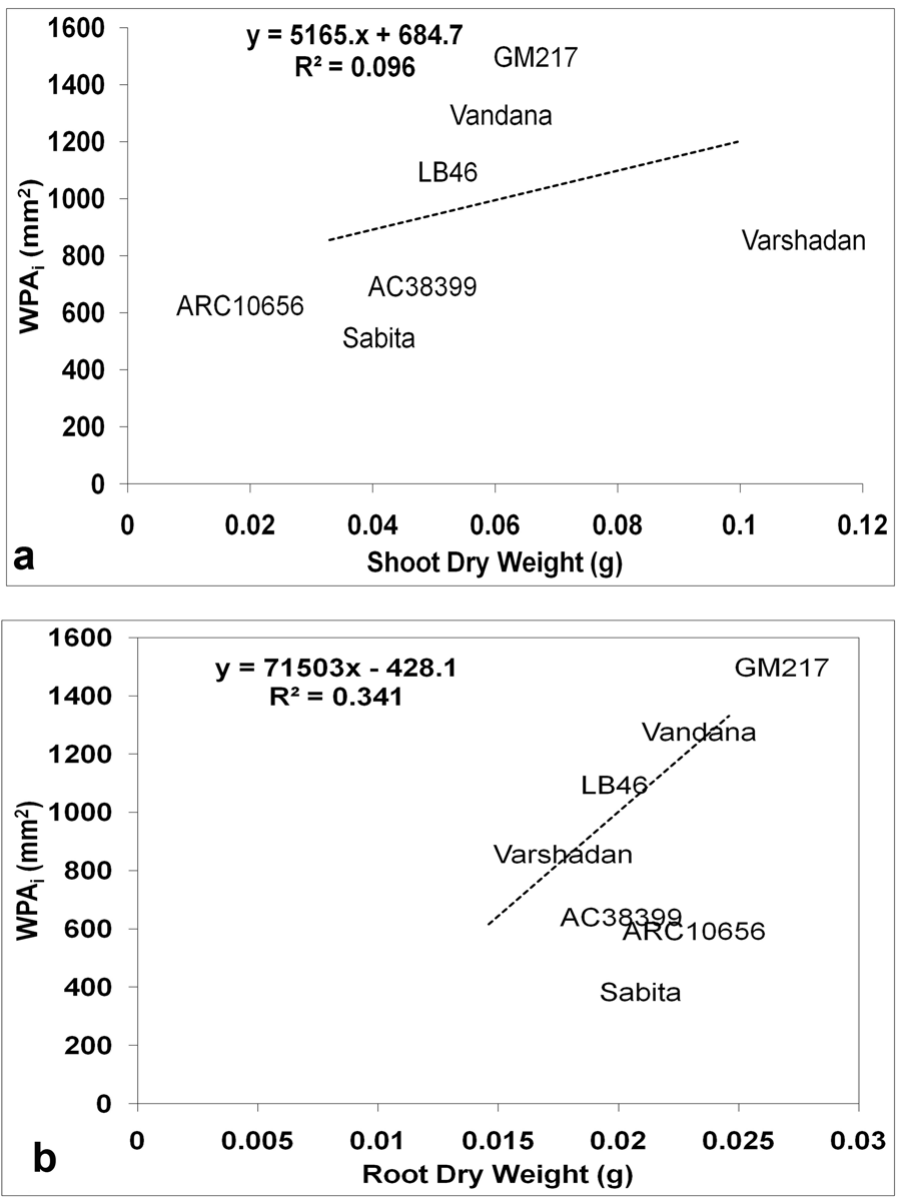
Result of linear regression analysis showing the medium association between morphological traits (shoot and root dry weight) and WPA_i_ of 14-day-old seedlings. **a** Shoot dry weight vs WPA_i_, **b** Root dry weight vs WPA_i_. The line indicates the fitted results representing the relationship between morphological traits and WPA_i_, *WPA*_*i*_ whole-plant area by non-destructive imaging

Data observed by RGB imaging have identified subtle differences between genotypes at both dates (14 and 28 DAS) of observation, while the same could not be achieved with the traditional way of measurement observed with respect to 14 DAS (Tables 1, 2, 3, 4). This suggests that phenotyping by imaging would be a better technique to find differences in the early stage of growth, which is dynamic and delicate for manual handling. Using destructive sampling, based on a higher growth rate, genotypes with seedling vigor were identified and were compared and selected through WPA_i_. This comparison was made to understand the potential and accuracy of the measurements obtained from RGB imaging. Among the destructive methods, CGR and CGR-WPA_i_ for shoots (R^2^ > 74%) and roots (R^2^ > 45%) were strongly related and RGR of shoot and root dry weight with RGR-WPA_i_ had weak similarity in the selection of genotypes, whereas AGR and AGR-WPA_i_ had no similarity. Thus, the high magnitude of association for seedling vigor estimated from the biomass-based destructive method by CGR can be replaced with the CGR-WPA_i_ technique, which was our primary experimental objective. To find out the fidelity of the WPA_i_ method, the traditional way of sampling was adopted to estimate whole-plant area (WPA_s_) by scanning individual leaf blades and stems. In the regression analysis, WPA_s_ and WPA_i_ were strongly related (R^2^ > 83%) on both (14 and 28 DAS) observation dates. The relationship between them was very high and WPA_i_ can also be used in place of WPA_s_ (destructive) to estimate seedling vigor. Similarly, Hairmansis et al. [15] and Campbell et al. [35] have identified tolerant rice genotypes under salinity by capturing morphological and physiological responses by processing RGB images in a non-destructive way. They have proved the successful introgression of imaging techniques in high-throughput phenotyping. Further, they have explained the reliability of imaging techniques by the linear relationship between fresh weight and projected image area. However, the dry weight of samples would always be preferred over the fresh weight of samples to avoid variability in moisture content among the samples and genotypes. Therefore, to comprehend the fidelity of imaging techniques, the relationship between WPA_i_ and shoot dry biomass was studied. A strong correlation existed between WPA_i_ and shoot dry weight at 28 DAS (r = 92, p < 0.01; R^2^ = 86%) and medium association at 14 DAS (r = 0.31; R^2^ = 9.6%). It is optimistic that accuracy might increase with the age of the seedlings with more biomass. Therefore, shoot area by images would be a good surrogate for estimating seedling vigor using shoot biomass up to 4 weeks of age (Fig. 6a, b) and, further, to determine the relationship between WPA_i_ and root dry weight. WPA_i_ of shoot and root dry weight was correlated and we could find a strong association between them at 28 DAS (r = 86, p < 0.01; R^2^ = 74%) and 14 DAS (r = 0.58; R^2^ = 34%). This suggests that WPA obtained by RGB imaging would also be useful for understanding the growth rate of below-ground parts of rice seedlings in relative time [36].

### Grouping of traits across DAS to determine genotypes with higher variability

**Fig. 7:**
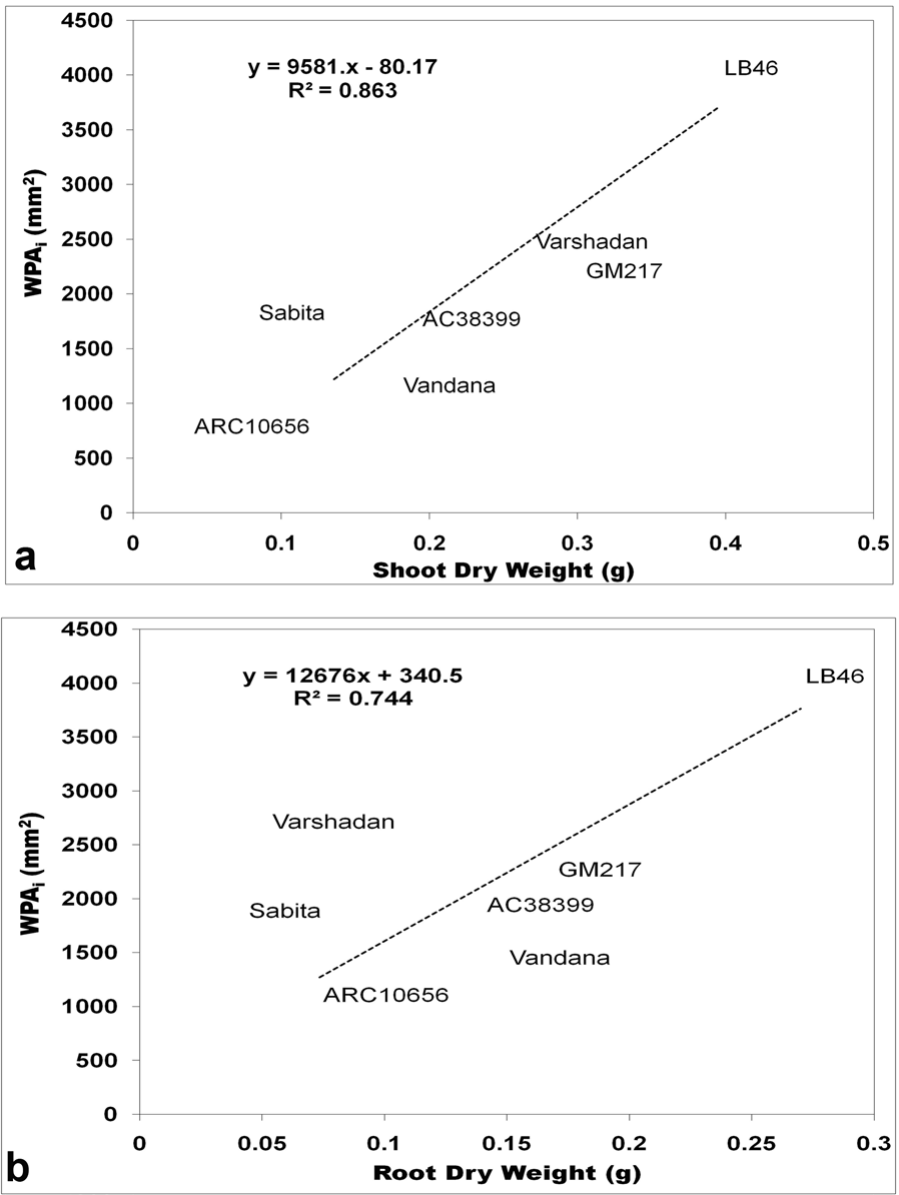
Result of linear regression analysis showing a strong correlation between morphological traits (shoot and root dry weight) and WPA_i_ of 28-day-old seedlings. **a** Shoot dry weight vs WPA_i_, **b** Root dry weight vs WPA_i_. The line indicates the fitted results representing the relationship between morphological traits and WPA_i_, *WPA*_*i*_ whole-plant area by non-destructive imaging

PCA was carried out for all seven genotypes to identify trends among the genotypes and the traits responsible for the source of variation for seedling vigor. Nine highly variable traits on day 14 and 10 traits on day 28 govern the highest variation among the seven genotypes. Across the two observation dates, the traits WPA_i_, WPA_s_, maximum leaf growth at 28 DAS (6th and 7th leaf area), convex hull, and top view area of both observation dates delivered the highest variation among 73 seedling traits (29 traits at 14 DAS and 44 traits at 28 DAS) (Fig. 7a, b). Thus, for the image-based measured parameters of whole-plant area, convex hull captured the maximum variability (R^2^ = 0.25 (14 DAS), R^2^ = 0.79 (28 DAS)), which has maximum variation toward WPA_i_ and is considered as an important trait for the selection of genotypes for seedling vigor. Thus, these traits were highly variable and contributed to the selection of genotypes for early seedling vigor and are thought to be useful in improving seedling vigor in rice through automated image phenotyping. Among the traits studied at two different times, the traits measured at 28 DAS contributed much to differentiate genotypes. Therefore, for the study involved in the identification of vigorous genotypes at the seedling stage, the parameters observed at 28 DAS by image-based phenotyping would be adequate. This would help in circumventing destructive sampling, thus saving resources, time, and labor involved in observing data at two different times. The present methodology in combination with the automated handling system would efficiently screen the dynamic responses of breeding lines in limited time. Further, this will help us to understand the mechanisms involved in enhancing the growth rate and genes to design a breeding program.

### Magnitude of component traits in determining vigorous genotypes

The digital color image data were used for estimating WPA_i_ and had a relationship with the component traits of seedling vigor, such as third leaf length (77%), third leaf area (51.29%), and stem area (61.33%) at 14 DAS, which explained the highest variation for WPA_i_. Whereas, at 28 DAS, fifth and sixth leaf area (77.79% and 53.25%, respectively), stem weight (82.30%), and tiller number (79.80%) had a higher magnitude of regression for WPA_i_. Leaf area at both 14 and 28 DAS seems to be a major determinant to predict genotype performance; in particular, the recent fully expanded leaves were found to have a higher contribution. This is in line with Hairmansis et al. [15] and Nguyen et al. [37], who reported that leaf traits would be the best predictor in identifying the performance of field pea and rice, respectively. This confirms that the physiologically active and fully expanded leaves could be used as representations to predict early vigor in rice. Shoot length at 14 and 28 DAS had a different level of contribution toward WPA_i_. Variation in shoot length at 14 DAS (0.95, p < 0.01) had a greater contribution toward WPA_i_ than shoot length at 28 DAS (− 0.19 ^ns^) and vice versa in the case of geometric traits observed by image analysis (caliper length, eccentricity, convex hull, and top view area). This might be due to the variation for shoot length expressed by genotypes at 14 DAS that was significantly different at 1% (Tables 1, 2), while at 28 DAS it had significance at 5%.

Understanding the association between traits would help to identify target genotypes with the surrogate traits. Length, width, and area of the leaf at each level made a different contribution toward WPA_i_ in determining vigorous genotypes. Third leaf length at 14 DAS had a positive relationship with WPA_i_ and explained 77.64% of WPA_i_ variation in comparison with early formed leaves (first and second leaf). The weak association of first and second leaf length with WPA_i_ might be due to their tiny nature and their decreased visibility in the image. On the other hand, the increase in leaf area of early formed leaves (first and second) had a positive correlation with root length. In the present experiment, leaf number had a strong positive association with biomass. The rise in the number of leaves in the early stage of seedling growth would be due to an increase in tiller number; ultimately, that would increase biomass. Further, an increase in leaf number would have a cascade positive effect on top view area, caliper length, and convex hull. The enhanced convex hull area increases ground cover by the canopy, which will have ample access to water and fertilizer. The existence of temporal and genotypic differences in canopy cover is considered an important trait for distinguishing genotypes with early vigor [38]. Good ground cover increases the weed smothering effect, provides ample access to fertilizer and water [39], and decreases loss of water by covering the ground. However, the increase in leaf number had a negative association with compactness and leaf width. This is in line with Richards [40]: the high rate of canopy cover was associated with an increased rate of tillers rather than leaf expansion. According to the ideal plant type concept, the plant should be compact in nature with few productive tillers and broad leaves. The ideal plant type concept would be more suitable for an ecosystem in which there is no weed competitiveness. The same concept would not be appropriate for an ecosystem in which weed competitiveness is a regular phenomenon, as in DSR. Therefore, a genotype with early seedling vigor accumulating high biomass and having enhanced convex hull and evenly spaced leaves with minimum compactness would be more suitable. A compact plant type would always have overlapped leaves with a 45-degree angle to avoid shade effects for more light interference. Genotypes with a better convex hull and evenly spaced narrow leaves with decreased leaf width and decreased shade effects for the contemporary leaves for proper light interference to improve photosynthesis would be preferred. Therefore, these geometric traits might play a big role in studying the architecture of the plant.

At 14 DAS, GM-217 and Vandana attained maximum growth with more leaves and increased shoot length and biomass. The high biomass and leaf number had increased the WPA_i_, top view area, eccentricity, and convex hull. This had allowed the genotypes to secure the top position while their growth rate from 14 to 28 DAS slowed. Genotypes LB-46 and Varshadhan had gained a pronounced growth rate by increasing tiller number. The increase in tiller number eventually increases leaf number, biomass, WPA_i_, top view area, convex hull, caliper length, and leaf area of terminal leaves. This helps the genotypes to be more vigorous at 28 DAS. The increase in top view area and convex hull enhances ground cover. Thus, the enhanced soil cover improves the weed smothering effect. Further, the increase in the size of the canopy (caliper length) and leaf area augments ground cover. These types of geometric trait data are difficult to generate through manual systems and are time-consuming [9].

A comparative study was done between the destructive and non-destructive methods to identify genotypes having high seedling vigor. Overall, LB-46 was found to be a common genotype across the methods used to judge the genotypes, with the highest seedling vigor. Varshadhan and AC38399 were identified as the next best genotypes by WPA_i_, while the traditional method identified GM-217 and Varshadhan as the next best performers. The variability in the position of genotypes between WPA_i_ and the traditional method might be due to the handling of different plant samples of the same variety at a relative time. In addition, their growth rate and manual handling of 2-week-old small seedlings would cause some errors. Therefore, the image analysis technique was found to be a very effective determinant of seedling vigor without human interference. These variations were easily captured through WPA_i_ and chances of human error could be decreased in such cases. Further, the traditional way of estimating vigor in the case of often and highly cross-pollinated crops would not be more reliable. Therefore, WPA_i_ has quite a few advantages and can be used at any stage of seedlings across different crops. On the other hand, we found some minor differences in the ranking of genotypes at 14 and 28 DAS between WPA_i_ and WPA_s_. These differences in the ranking of genotypes between imaging and scanning might be due to the overlapping of leaves during imaging and some unexposed area of droopy leaves.

## Methods

### Plant materials

Seven rice (*Oryza sativa* L.) genotypes of improved and traditional lines, LB-46, GM-217, AC38399, ARC10656, Vandana, Sabita (NC492), and Varshadhan, were used in this study. Varshadhan and Sabita were developed at the International Rice Research Institute (IRRI), Philippines, and in Chinsurah, West Bengal, India, respectively, for the semi-deep ecosystem. Vandana was developed for upland conditions by ICAR-National Rice Research Institute (NRRI). ARC10656 and GM-217 belong to traditional rice collections of Assam and Tamil Nadu, respectively. LB-46 was the progeny of *Oryza sativa* x *O. nivara*, developed by NRRI, Cuttack.

### Seed selection and sterilization

Seeds of all the genotypes were grown and harvested in the wet season of 2017 and packed separately for drying. All the genotypes were sorted by uniform seed size and underwent heat treatment to break seed dormancy. The seeds were kept in a hot-air oven at 50 °C for 45 h. Later, the seeds were surface-sterilized with 75% ethanol for 1 min. These seeds were further sterilized with 2.5% sodium hypochlorite for 20 min and washed five times with sterile distilled water to remove any traces of sterilizing agent.

### Growing conditions and experimental design

Five seeds of each genotype were sown into a pot (white color, 20 cm height × 15 cm diameter) containing 2.5 kg of clayey loam without any external fertilizer. Each genotype was raised in five pots with five biological replicates. The spacing between plants was maintained at 20 × 15 cm to simulate the recommended spacing for direct-seeded conditions. Therefore, pots were arranged in such a way to have 20 cm between rows and 15 cm within rows. After one week, the seedlings were thinned to a single seedling per pot by maintaining sufficient moisture with a proper drainage hole at the base. The experiment was conducted in the net house at NRRI (20°27′09″ N, 85°55′57″ E, 26 masl), Cuttack, during March 2018. The plants received 13 h/11 h of day/night cycle. The average temperature in the net house was 33 °C in the day and 23 °C at night, with an average light intensity of ~ 1200 µmol m^−2^ s^−1^ during the observation period.

### Image capture and analysis

At 14 and 28 DAS, images of five biological replicates per genotype were captured, and the same set of plants was used to measure the morphometric data to estimate growth parameters on the same day. Three different techniques were followed to measure the morphometric data of the whole plant: (1) non-destructive imaging and (2) two versions of destructive growth analysis.

### Method-1: Non-destructive imaging

**Fig. 8:**
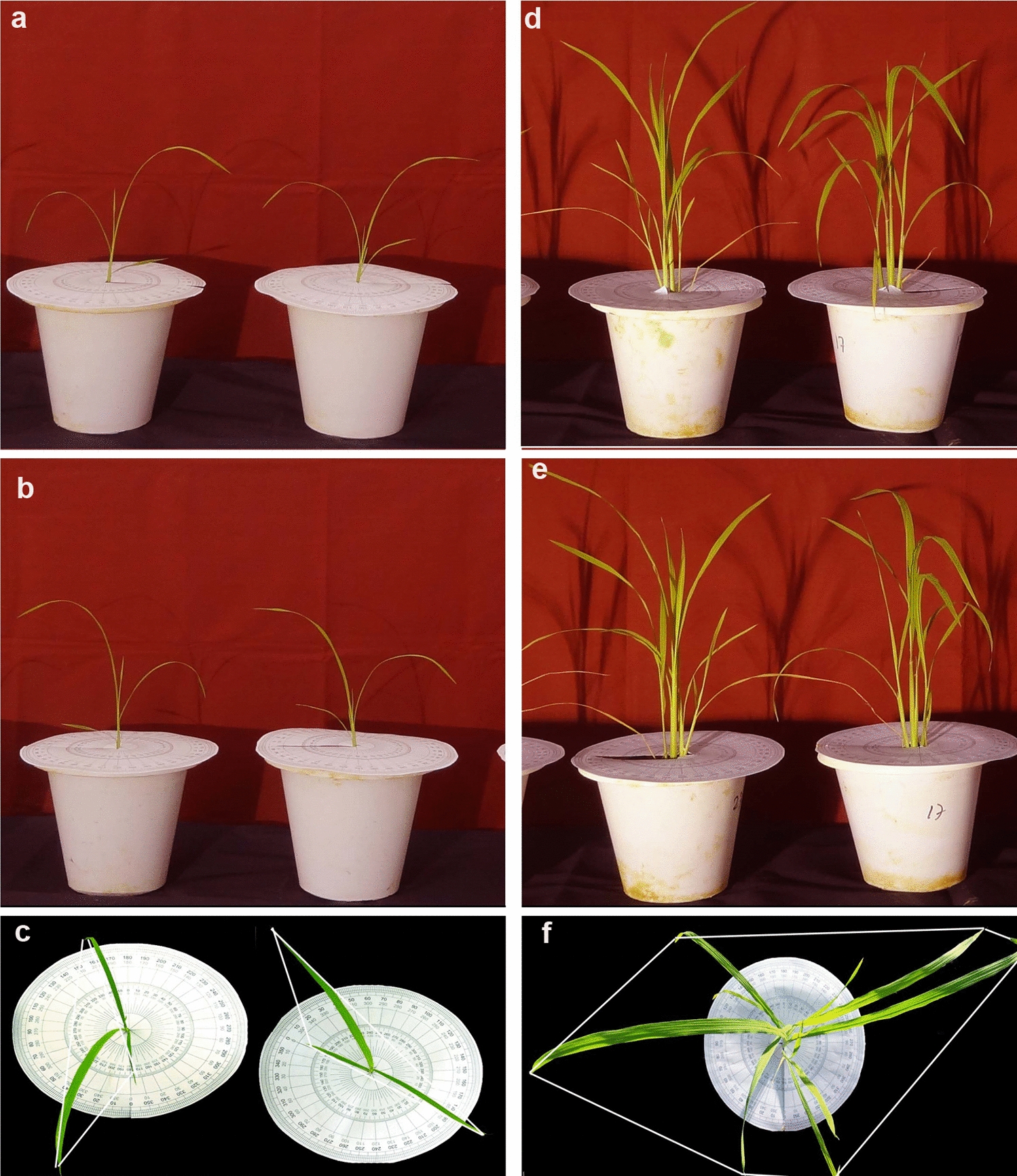
Representative images of cv. LB-46 were taken at 14 and 28 days after sowing (DAS). **a**, **d** Front (side) view of RGB images at 14 and 28 DAS, respectively. **b**, **d** Rear (opposite) view of RGB images of the same plants shown in **a**, **d**. **c**, **f** Top view RGB images at 14 and 28 DAS, respectively. Convex hull, white line enclosing plants (**c**, **f**)

In the first method, data were observed non-destructively on a whole-plant basis using a 12-megapixel Nikon camera (RGB images) at a distance of 1.5 m. To have a uniform background, the potted plant was placed over a raised platform having a dark background behind and over the platform. High-intensity artificial light was used to obtain a uniform background effect and strong wind or airflow was checked to avoid movement of leaves during imaging. A known scale indicator/ruler was placed above and adjacent to the pot to calculate the leaf/whole-plant area with proper labeling. Three colored images per plant were captured from the top of the plant and two from either side of the plant at 90° (Fig. 8). A uniform distance between plant and camera, camera setting, and background light were maintained throughout the imaging process.

**Fig. 9:**
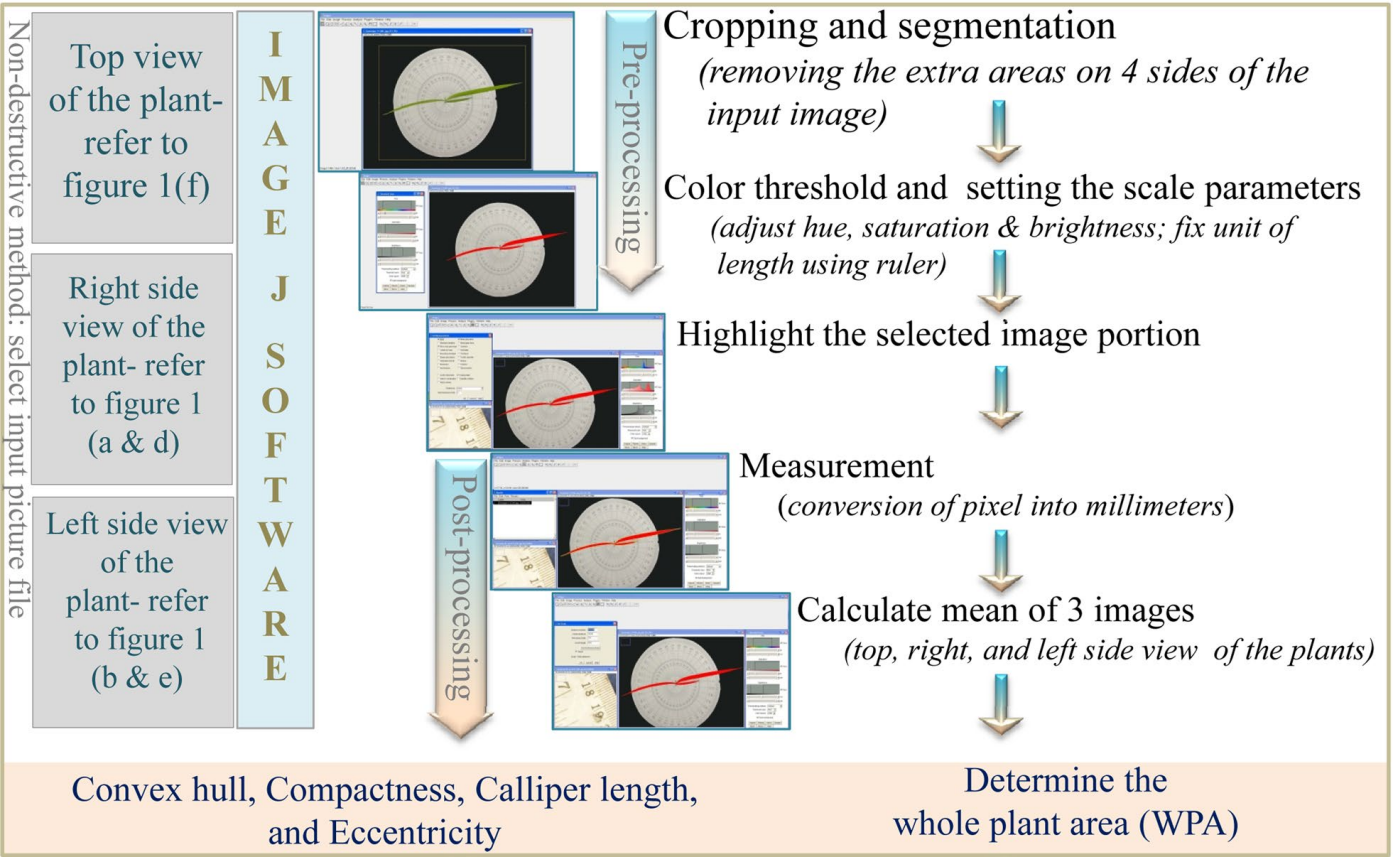
The workflow illustrates the steps involved in the phenotyping of early seedling vigor (ESV) in rice. The images (RGB) of the whole plant in three different views were captured through a 12-megapixel Nikon camera and were processed using Image J software. Each of the images taken from one top view and the other two side views was stored in JPEG format. These images were uploaded into Image J and followed by image pre-processing, which includes image cropping, maintaining the threshold level of color intensity and adjustment of brightness, and setting the scale measurements. Image post-processing was involved mainly in selecting the desired portion of the plant area and converting it into a binary scale of color to establish a specific value. The selected portion of the image area was converted from pixels to square millimeters. Following that, the summary of three images (top and two side views) of crop area was used to estimate the whole-plant area (WPA) and expressed in square millimeters. These overall steps of pre- and post-processing of each image were followed to calculate WPA. Other than WPA, the top view image was used for calculating geometric measurements such as convex hull, compactness, caliper length, and eccentricity

The recorded images were processed using Image J software. In brief, the respective image was cropped to remove any excess area on all four sides by retaining the potted plants. To assess the green portion of the plant, the cropped image was used to separate the plants from the imaging background by selecting the color threshold. To identify the plant as a whole and to remove any further noise, all portions of the plant were highlighted using hue, saturation, and brightness (Fig. 9). Using the known indicator, the whole-plant area/desired portion of the plant was converted from pixels to square millimeters. The summed area of all three images (top and two side views) was used to estimate the whole-plant area (WPA_i_) and expressed in square millimeters. Additionally, geometric traits such as convex hull, compactness, caliper length, and eccentricity were determined from the top view image (captured from the top of the plant) as described by Neilson et al. [9]. The observations collected from imaging were used to calculate relative (RGR_i_), absolute (AGR_i_), and crop (CGR_i_) growth rate of the plants. The growth rate was calculated using the average of final and initial WPA_i_, simulating the way the growth rate was calculated for the destructive method.

$$\begin{aligned} {\text{RGR}}_{{\text{i}}} & = \left( {\log _{{\text{e}}} {\text{A}}_{2} - \log _{{\text{e}}} {\text{A}}_{1} /{\text{t}}_{2}-{\text{t}}_{1} } \right)\;{\text{mm}}^{2} {\text{day}}^{{ - 1}} \\ {\text{AGR}}_{{\text{i}}} & = \left( {{\text{A}}_{2} - {\text{A}}_{1} /{\text{t}}_{2} - {\text{t}}_{1} } \right)\;{\text{mm}}^{2} {\text{day}}^{{ - 1}} \\ {\text{CGR}}_{{\text{i}}} & = \left( {{\text{A}}_{2} - {\text{A}}_{1} } \right)/{\text{P}}\left( {{\text{t}}_{2} - {\text{t}}_{1} } \right)\;{\text{mm}}^{2} {\text{m}}^{{ - 2}} {\text{day}}^{{ - 1}} \\ \end{aligned}$$

where A_1_ and A_2_ are the whole-plant area at times t_1_ and t_2_, respectively, i = image-based, log_e_ = natural logarithm, and P = spacing (m^2^).

### Method-2 and -3: Destructive growth analysis

The plants used for imaging were uprooted and the roots were washed to record morphometric traits. This was followed by the same plants being used for the destructive method. The leaves were meticulously cut from the stem and placed between the paper pages of a clean notebook to maintain their shape. Later, the leaves and stem were scanned using a flatbed scanner with a ruler to calculate the leaf/whole-plant area using Image J software. The scanned images were used to estimate the area of each leaf and stem following the steps adapted to convert pixels to square millimeters as described earlier in the section on image capture and analysis. Traits such as tiller number, number of leaves, stem thickness (mm), shoot length (mm), root length (mm), shoot dry weight (g), root dry weight (g), specific leaf weight (g), and stem weight (g) were manually observed, while specific leaf length (mm), width (mm), and area (mm^2^) and stem area (mm^2^) were measured from scanned images of the flatbed scanner by destructive sampling. The summed area of all leaves and stems observed from the scanned images was used to estimate the whole-plant area (WPA_s_). In addition, the area of a single leaf blade calculated from the scanned image was compared with the biomass of the respective leaf and WPA_i_ to understand the relationship and percentage of variation contributed by them toward WPA_i_.

All three methods were compared to assess the fidelity of the data obtained through the imaging process. To assess the growth rate of seedlings, absolute growth rate, relative growth rate, and crop growth rate were calculated accordingly:

$${\text{AGR}}_{\text{m}} = \left( {{\text{h}}_{ 2} {-}{\text{h}}_{ 1} } \right)/\left( {{\text{t}}_{ 2} {-}{\text{t}}_{ 1} } \right)\;{\text{mm day}}^{ - 1}$$

where h_1_ and h_2_ are plant height at times t_1_ and t_2_, respectively, and m = manual method.

RGR was determined by using the dry weight of periodical observations and represented as mg g^−1^ day^−1^.

$${\text{RGR}}_{{\text{m}}} = \left( {\log _{{\text{e}}} {\text{W}}_{2} - \log _{{\text{e}}} {\text{W}}_{1} } \right)/\left( {{\text{t}}_{2} - {\text{t}}_{1} } \right)$$

where W_1_ and W_2_ are plant dry weights at times t_1_ and t_2_, respectively.

CGR was calculated by measuring plant dry weight at a regular interval of time divided by land area and represented as g m^−2^ day^−1^:

$${\text{CGR}}_{{\text{m}}} = \left( {{\text{W}}_{2} - {\text{W}}_{1} } \right)/{\text{P}}\left( {{\text{t}}_{2} - {\text{t}}_{1} } \right)$$

where W_1_ and W_2_ are plant dry weights at times t_1_ and t_2_, respectively, and P = spacing (m^2^).

Linear regression was estimated between WPA_i_ and seedling traits using MS Office Excel 2016. Principal component analysis (PCA) was performed with 29 traits at 14 DAS and 44 traits at 28 DAS to estimate the variability among genotypes and traits. Biplot figures explain the variances of the variables and correlation between the variables through vectors and similarity between genotypes in the multivariate space based on the nature of growth rate [41, 42]. These analyses were performed using Windostat 7.5 software.

## Conclusions

In the present experiment, the non-destructive-based imaging technique captured the dynamic responses of plants in the early stage and revealed significant differences across genotypes. Consistency in the ranking of genotypes across different methods and a strong correlation between morphological and image-associated traits confirm the reliability and reproducibility of the proposed method. The proposed imaging technique and the identified geometric traits convex hull and top view area were found to be significant in determining promising genotypes for early seedling vigor during the initial phase of plant establishment. Further, the method saves resources, time, and labor by validating that the parameters observed at 28 DAS are adequate in identifying vigorous genotypes. This has raised confidence that imaging techniques have the potential to identify and differentiate small differences that are considered as phenotypically identical and difficult to distinguish by unidentifiable traits through conventional measurements. To capitalize on the present non-destructive imaging technique as a high-throughput to handle more samples in a given time, the method needs to be fully automated. This would enable integrating the platform as a tool with the forward genetics approach in the identification of QTLs/genes for the traits identified under this system for possible future improvements in the study area of direct-seeded rice.

## Data Availability

The datasets during and/or analyzed during the current study are available from the corresponding author upon request.

## Abbreviations

AGR: absolute growth rate
CGR: crop growth rate
DAS: days after sowing
DSR: direct-seeded rice
PCA: principal component analysis
RGR: relative growth rate
WPA: whole-plant area
WPA_i_: non-destructive imaging
WPAs: destructive-flatbed scanner
